# Oxidative stress increases in liver of lactating rats after BPF-low-dose exposure: perinatal effects in the offspring

**DOI:** 10.1038/s41598-023-38434-w

**Published:** 2023-07-11

**Authors:** Beatriz Linillos-Pradillo, Lisa Rancan, Julio García Murias, Margret Schlumpf, Walter Lichtensteiger, J. A. F. Tresguerres, Elena Vara, Sergio D. Paredes

**Affiliations:** 1 Department of Biochemistry and Molecular Biology, School of Medicine, Complutense University of Madrid, Madrid, Spain; 2 Department of Physiology, School of Medicine, Complutense University of Madrid, Madrid, Spain; 3 GREEN Tox and Institute of Veterinary Pharmacology and Toxicology, University of Zurich, Zurich, Switzerland

**Keywords:** Environmental sciences, Endocrinology

## Abstract

Bisphenol F (BPF) is replacing Bisphenol A (BPA) in the manufacture of products due to endocrine-disrupting effects. BPF monomers can also be released into the environment and enter the food chain, resulting in human exposure to low doses. Since bisphenols are primarily metabolized by the liver, this organ is more vulnerable to lower doses of bisphenols than others. Exposure during prenatal development may increase the risk of diseases in adulthood. The aim was to evaluate whether BPF administration could generate oxidative stress in liver of lactating rats, and whether these effects may be also observed in female and male postnatal day 6 (PND6) offspring. Long Evans rats received oral treatment: Control, BPF-low-dose (LBPF) 0.0365 mg/kg b.w./day, and BPF-high-dose (HBPF) 3.65 mg/kg b.w./day. The levels of antioxidant enzymes (CAT, SOD, GR, GPx and GST), glutathione system (GSH, GSSG) and lipid damage markers (MDA, LPO) were measured using colorimetric methods in liver of both lactating dams and in PND6 offspring. Mean values were analyzed using Prism-7. LBPF affected liver defense mechanisms (antioxidant enzymes and glutathione system), increasing ROS levels and producing lipid peroxidation in lactating dams. Similar effects were found in female and male PND6 offspring as a consequence of perinatal exposure.

## Introduction

Numerous scientific studies describe the toxic effects of Bisphenol A (BPA), an endocrine disruptor widely used in industry. Its exposure is associated with adverse health effects (cardiovascular, respiratory, diabetes, renal, obesity and reproductive disorders)^1–3^ and its use is being restricted.

BPA is being replaced by allegedly safer analogues such as Bisphenol F (BPF, bis (4-hydroxyphenyl) methane). BPF is used in the manufacture of epoxy resins and coatings^4^ and in polymers that give materials increased thickness and durability. It is found frequently in plastic, varnish, dental sealant and personal care products and office paper^5,6^. Its presence in food packaging production and cans consumed in everyday life is very relevant^7^. In addition, there is environmental contamination with BPF, as it is found in household dust and in surface waters, sediments and sewage effluents^5,6^.

Exposure to this chemical occurs via three routes: oral, dermal and inhalation. BPF has been found in human urine samples in several European countries at concentrations comparable to BPA^8–10^, in breast milk^11^ and in serum^12^. It has been detected in human plasma at concentrations three times higher than BPA^13^. BPF is not only detected in adult tissues and fluids, but can also cross the blood–brain barrier and the placental barrier and reach the fetus^14^.

The impact of exposure to bisphenols is crucial in early life, therefore, there is a need to investigate the effect not only on adult organisms, but also the perinatal effect on offspring.

Previous studies reported that BPF may have a similar toxicity and mechanism of action to BPA, due to their structural and physicochemical similarities^7,15,16^. Li et al.^17,18^ have reported that loss of the antioxidant system leads to oxidative damage in the liver. Exposure to BPA is known to impair the antioxidant defense system, increases lipid peroxidation and causes oxidative damage to the liver^19,20^. Recent studies by our research group showed that low dose of BPA caused liver injury in lactating dams and had a perinatal effect in female PND6 offspring by increasing oxidative stress levels, triggering an inflammatory response and apoptosis pathways in the liver, the organ responsible for detoxification of this endocrine disruptor^21^. However, the mechanisms and effects of BPF on oxidative stress in the liver remain unclear.

The aim of this study was to evaluate the effects after exposure to two doses of BPF on oxidative stress: antioxidant enzymes, glutathione system and indicators of lipid damage in the liver of lactating dams. Moreover, it was studied whether this effect can also be observed in the liver of female and male offspring at postnatal day 6 (PND6).

## Results

The liver has an endogenous antioxidant defense system to prevent cell damage, consisting of antioxidant enzymes and the glutathione system.

**Figure 1:**
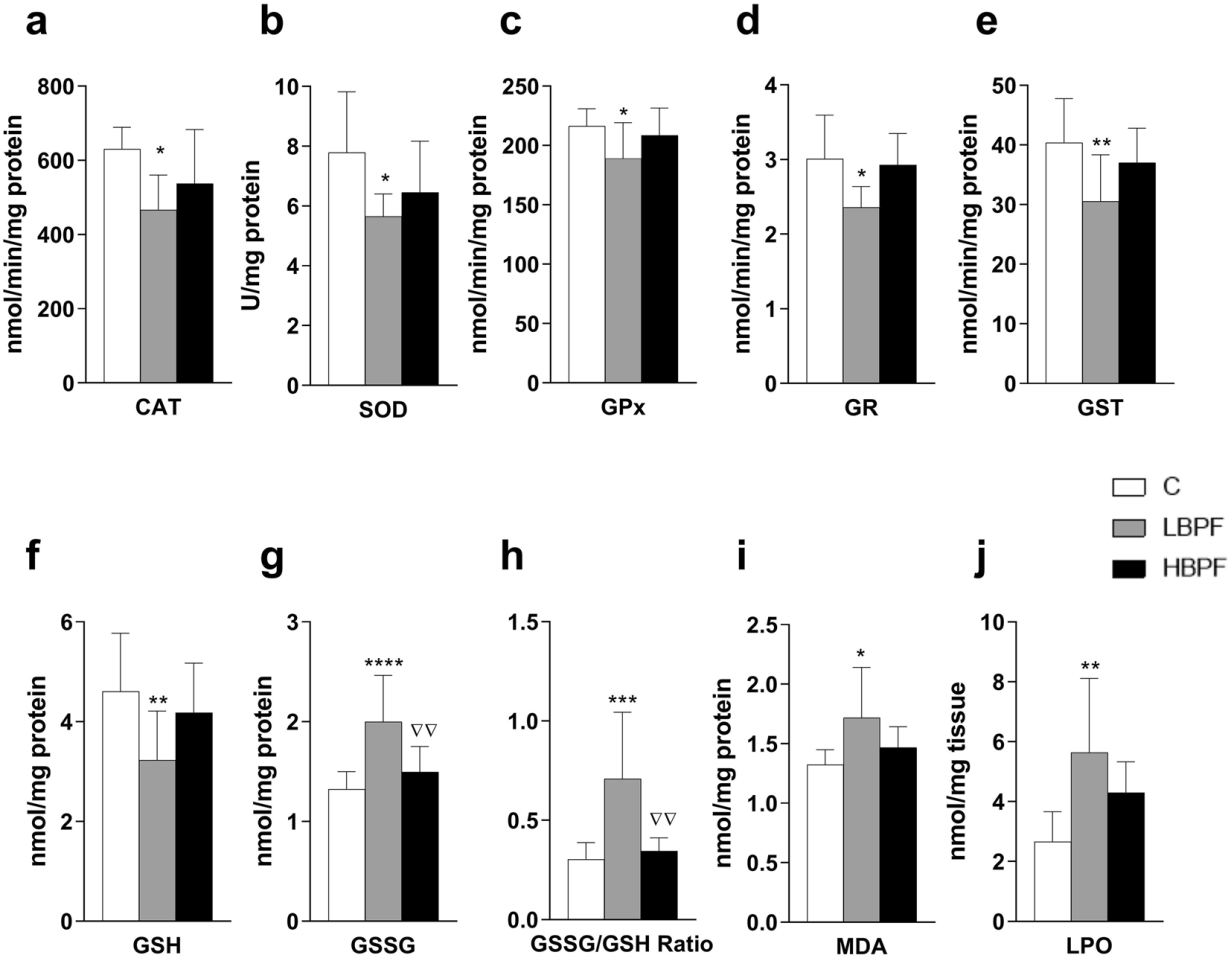
Effects of BPF administration on antioxidant enzymes, glutathione concentrations and oxidative stress biomarkers in liver from lactating dams. (**a**) Enzymatic activity of catalase (CAT) in nmol/min/mg protein; (**b**) Superoxide dismutase (SOD) in U/mg protein; (**c**) Glutathione peroxidase (GPx) in nmol/min/mg protein; (**d**) Glutathione reductase (GR) in nmol/min/mg protein; and (**e**) Glutathione S-transferase (GST) in nmol/min/mg protein. (**f**) Concentration of reduced glutathione (GSH) in nmol/mg protein; (**g**) Concentration of oxidized glutathione (GSSG) in nmol/mg protein. (**h**) GSSG/GSH ratio. (**i**) Malondialdehyde (MDA) content in nmol/mg protein. j) Lipid hydroperoxide (LPO) content in nmol/mg tissue. Data represent mean ± SD. n = 6 lactating control dams; n = 6 lactating LBPF dams; n = 10 lactating HBPF dams (two replicates for each sample). Statistical significance was determined by one-way ANOVA. *P < 0.05; **P < 0.01; ***P < 0.001; ****P < 0.0001 compared to Control group. ∇∇*P* < 0.01, LBPF vs. HBPF.

When lactating females were treated with LBPF, all antioxidant enzyme activities (CAT, SOD, GPx, GR and GST) were significantly decreased as compared to the control group (Figs. 1a–e respectively). No significant changes were shown in antioxidant enzymes in HBPF-treated dams.

Regarding glutathione concentrations, a decrease in reduced glutathione (GSH) concentration was observed in LBPF-treated dams (Fig. 1f). GSSG concentration increased significantly in LBPF-treated dams as compared to the control group and significant differences were also observed between both treatment groups, resulting in higher levels of oxidative stress in the LBPF group (Fig. 1g). The same was observed for the GSSG/GSH ratio, as a marker of oxidative stress, indicating a more marked redox imbalance after LBPF administration (Fig. 1h).

Oxidative stress produces free radicals that can easily react with cell membrane lipids, triggering the production of lipid peroxides. Also, a significant increase in MDA and LPO levels, two products used to measure oxidative lipid damage, was observed after LBPF administration in lactating dams (Fig. 1i and j).

To study whether perinatal administration of BPF was able to generate alterations of the oxidant/antioxidant balance, we evaluated the same parameters in the liver of female and male offspring.

**Figure 2:**
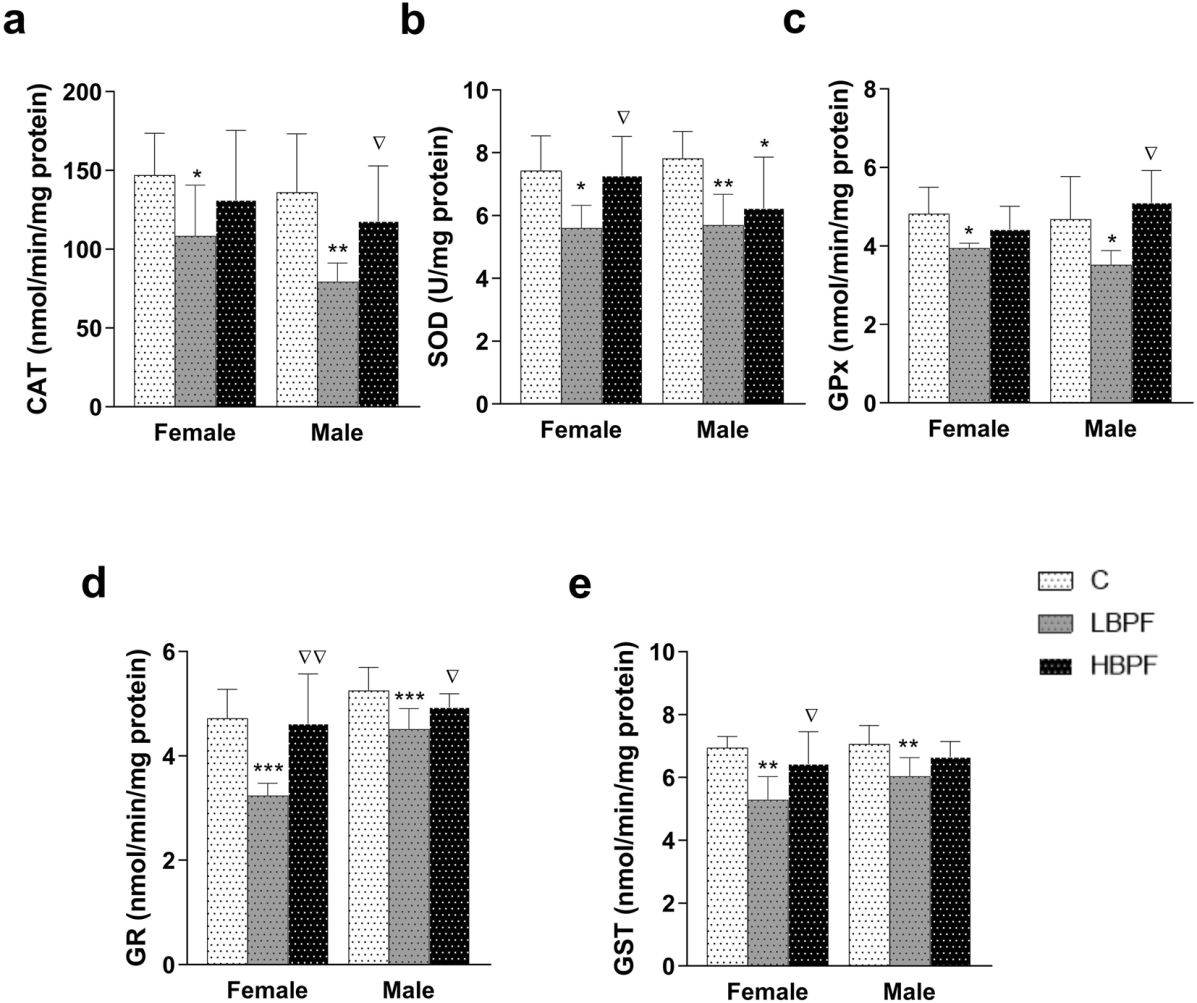
Effects of BPF administration on antioxidant enzymes in liver from female and male PND6 offspring. (**a**) Enzymatic activity of catalase (CAT) in nmol/min/mg protein; (**b**) Superoxide dismutase (SOD) in U/mg protein; (**c**) Glutathione peroxidase (GPx) in nmol/min/mg protein; d) Glutathione reductase (GR) in nmol/min/mg protein; and (**e**) Glutathione S-transferase (GST) in nmol/min/mg protein. Data represent mean ± SD. n = 12 female PND6 pups; n = 12 male PND6 pups for each experimental group with two replicates for each sample (control, LBPF and HBPF). Statistical significance was determined by one-way ANOVA. *P < 0.05; **P < 0.01; ***P < 0.001 compared to Control group. ∇*P* < 0.05; ∇∇*P* < 0.01, LBPF vs. HBPF.

When female PND6 offspring were perinatally exposed to LBPF, all antioxidant enzyme activities (CAT, SOD, GPx, GR and GST) were significantly decreased as compared to the control group (Fig. 2a–e). Significant changes were observed between both treatment groups with respect to the activities of the antioxidant enzymes SOD, GR and GST, with the levels in LBPF-exposed female offspring significantly lower than those observed in the HBPF group (Fig. 2b, d and e respectively).

Similar effects were found in male offspring exposed perinatally to BPF, i.e., a decrease in the activity of all antioxidant enzymes (CAT, SOD, GPx, GR and GST) (Fig. 2a–e). Regarding CAT, GPx and GR levels, significant changes were observed between both treatment groups, being the levels in LBPF-exposed male offspring significantly decreased as compared to the HBPF-exposed group (Fig. 2a, c and d). There was also a decrease in SOD levels in comparison to the control group in male offspring after perinatal exposure to HBPF (Fig. 2b).

**Figure 3:**
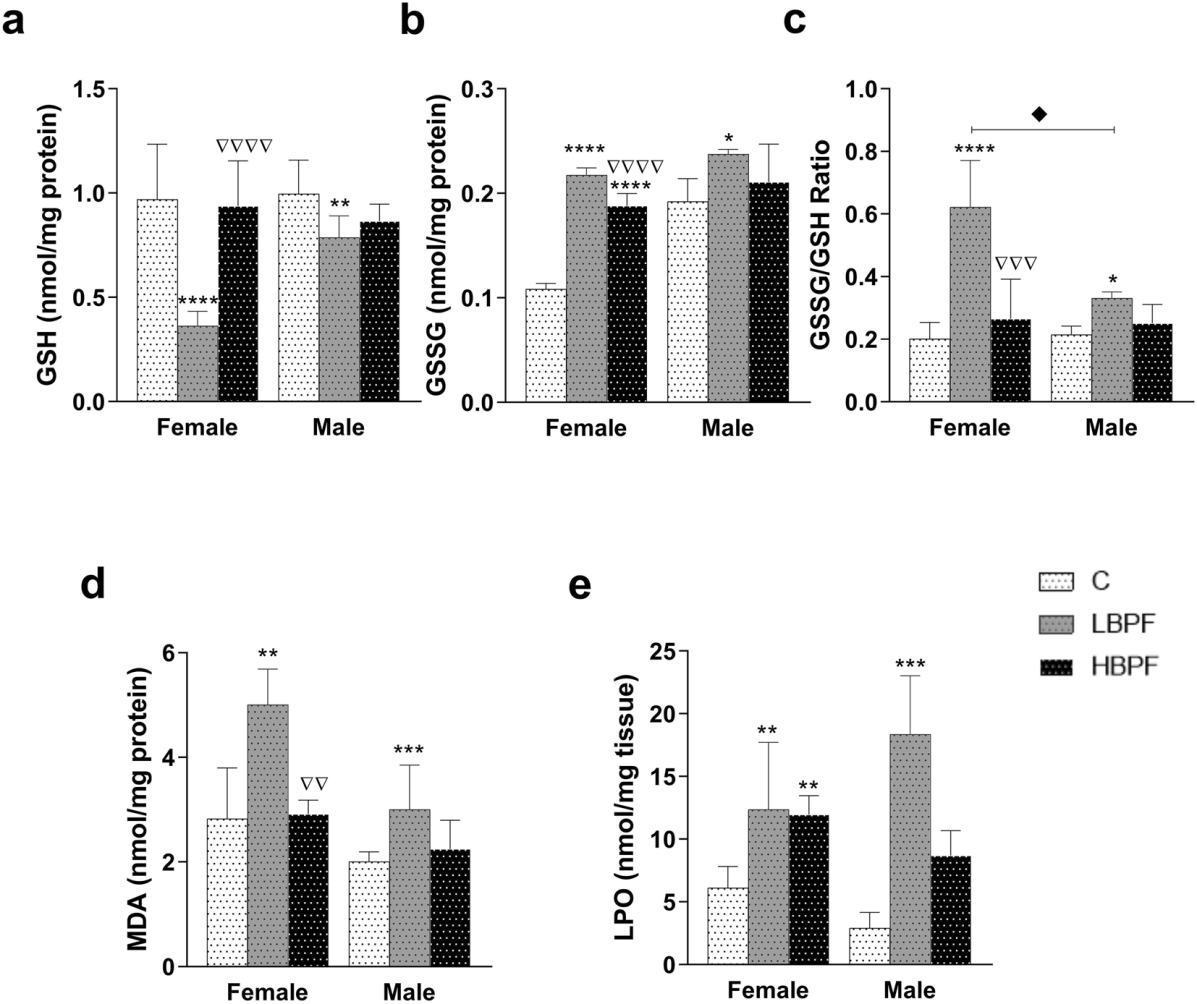
Effects of BPF administration on glutathione concentrations and oxidative stress biomarkers in liver from female and male PND6 offspring. (**a**) Concentration of reduced glutathione (GSH) in nmol/mg protein. b) Concentration of oxidized glutathione (GSSG) in nmol/mg protein. (**c**) GSSG/GSH ratio. (**d**) Malondialdehyde (MDA) content in nmol/mg protein. e) Lipid hydroperoxide (LPO) content in nmol/mg tissue. Data represent mean ± SD. n = 12 female PND6 pups; n = 12 male PND6 pups for each experimental group with two replicates for each sample (control, LBPF and HBPF). Statistical significance was determined by one-way ANOVA. *P < 0.05; **P < 0.01; ***P < 0.001; ****P < 0.0001 compared to Control group. ∇∇*P* < 0.01; ∇∇∇*P* < 0.001, ∇∇∇∇*P* < 0.0001, LBPF vs. HBPF. ♦ LBPF Female vs. LBPF Male.

Regarding the glutathione system, a decrease in reduced glutathione (GSH) concentration (Fig. 3a) and an increase in oxidized glutathione (GSSG) concentration (Fig. 3b) were observed in LBPF-exposed female offspring. An increase in the GSSG/GSH ratio in LBPF-exposed female offspring was also observed (Fig. 3c). These three parameters showed significant differences between LBPF and HBPF treatments, with more noticeable effects in the LBPF group. There was also an increase in GSSG levels in female offspring after perinatal exposure to HBPF compared to the control group (Fig. 3b); with no significant changes in GSH levels.

When PND6 male offspring were perinatally exposed to LBPF, a decrease in GSH levels, an increase in GSSG and thus an increment in the GSSG/GSH ratio was observed compared to the control group (Fig. 3a–c). When comparing both sexes, it was noteworthy that the GSSG/GSH ratio, a marker of oxidative stress, was much higher in female offspring; therefore, female offspring showed higher levels of GSSG and thus a greater imbalance in the glutathione system (Fig. 3c). This elevation was also observed in LBPF-treated dams (Fig. 1h).

Decreased levels of antioxidant enzymes and alteration of the glutathione system increased MDA and LPO levels in both LBPF-exposed female and male offspring (Fig. 3d and e). In addition, there were significant differences in MDA levels between LBPF- and HBPF-exposed females, with higher levels after LBPF exposure (Fig. 3d). After perinatal exposure to HBPF, there was also an increase in LPO levels in female offspring as compared to the control group (Fig. 3e).

## Discussion

Due to the widespread use of BPF as an alternative to BPA, residues of BPF can migrate into food and BPF monomers have been found in canned foods and soft drinks with an average concentration of 0.18 mg/dL^4,22,23^. In a previous study with female Sprague–Dawley rats that were gavaged with a single dose of BPF (7 and 100 mg/kg/b.w.), it was shown that excretion of BPF residues occurred mainly in urine via BPF-conjugated sulfate. However, BPF residues were detectable in all tissues examined at 96 h, with the highest amounts found in the liver (0.5% of the dose). The liver is an essential organ for detoxification and metabolism, being more especially vulnerable to damage^14^.

For this reason, in this study the effects of BPF administered by the oral route at two concentrations: low dose of 0.0365 mg/kg b.w./day (LBPF) and high dose of 3.65 mg/kg b.w./day (HBPF) in the liver of lactating dams were evaluated. Also, the perinatal effect in female and male PND6 offspring after BPF exposure was studied.

Oxidative stress represents the imbalance in the production and removal of reactive oxygen species (ROS) and can be considered a pathological mechanism that contributes to the initiation and development of liver injury. To respond to oxidative stress under physiological conditions, the liver has both an enzymatic antioxidant system and the glutathione system^17^.

The first line of defense against cell attack by ROS are the enzymes CAT and SOD. SOD carries out the dismutation of superoxide anion radicals into hydrogen peroxide and molecular oxygen. This hydrogen peroxide is degraded by CAT to form water and oxygen allowing the unsaturated fatty acids of the cell membrane to be protected from peroxidation. In the present study, a decrease in the enzymatic activity of CAT and SOD after administration of LBPF in lactating dams was observed^24^.

GSH is an antioxidant molecule that metabolizes and detoxifies xenobiotics that are directly conjugated to protect the cells from oxidative damage. Reduced GSH levels were found after exposure to LBPF-treated dams. This GSH is used as a cofactor by the antioxidant enzyme GPx which catalyzes the degradation of hydroperoxides into hydroxyl compounds. The activity of GPx is closely related to its cofactor, GSH, and after observing GSH depletion, it can lead to a decrease in the activity of this enzyme in liver cells, as observed the present investigation. Oxidized glutathione (GSSG), produced after reduction of an organic hydroperoxide by GPx, is recycled to its reduced state by GR and NADPH. In this study, we observed a decrease in the enzymatic activity of GR and an excess of GSSG concentration, more noticeable after administration of LBPF as compared to the control and HBPF groups in lactating dams^19,25^. GST is a phase II detoxifying enzyme with a critical role in cellular protection against ROS and toxic xenobiotics; it catalyzes the conjugation of GSH in reaction to endogenous and exogenous electrophiles. The activity of GST was decreased in LBPF-treated dams, impairing the correct elimination of this xenobiotic in the liver^19,26^.

Therefore, in our study, LBPF caused oxidative damage in liver cells resulting in decreased antioxidant enzyme activities and alteration of the glutathione system. This was manifested by an increased GSSG/GSH ratio, a marker of oxidative stress. Also, this ratio was also statistically significant after comparison of both doses of BPF, with higher levels implying more intense actions of LBPF found in the liver of lactating dams.

Increased ROS also acts on macromolecules such as polyunsaturated fatty acids to initiate lipid peroxidation, as well as changing cell membrane fluidity and permeability. In our results, reduced antioxidant enzyme activity contributed to increased levels of MDA and LPO, two end products of lipid peroxidation used to monitor cell membrane damage in the liver of LBPF-treated dams^27^.

Our results are consistent with previous research following in vivo and in vitro BPF exposure, in liver and other organs, using different animal species. The cytotoxic effects of seven concentrations of BPF during 24 h were studied on hepatocytes isolated from the liver of rainbow trout. CAT activity was decreased with all BPF concentrations. Also, GSH content was reduced with the highest concentration of BPF, and MDA content was increased significantly at BPF concentrations between 15.63 and 250 μM^19^. In addition, BPF exposure induced NAFLD-like changes (non-alcoholic fatty liver disease), with obvious lipid droplet deposition, triglyceride (TG) and fatty acid increases in mouse livers administered with BPF (50 mg/kg/day) for 30 days via subcutaneous injection^28^. In the study by Higashira et al.^29^, carried out in rats orally gavaged with different concentrations of BPF (0, 20, 100, and 500 mg/kg per day) during 28 days, BPF caused liver toxicity based on clinical biochemical parameters and liver weight, but without histopathological changes. In a recent study by Sun et al.^30^, they indicated altered liver function by BPF exposure in male mice following increased hepatic ALT and increased plasma AST levels after low doses of BPF. After exposure to BPF at 100 mg/kg in the reproductive tissues of male rats, CAT and SOD activities decreased and increased ROS levels and lipid peroxidation^27^. Maćczak et al.^31^ found depleted GSH levels in human erythrocytes exposed to BPF and increased levels of lipid peroxidation. In vitro study on KGN cells, showed that BPF exhibited slight toxic effects and increased the damage to biomacromolecules (MDA, 8-OHdG) after BPF exposure^32^. Furthermore, the exposure of BPF resulted in higher MDA contents in the larvae of zebrafish and finally led to apoptosis^33^. Furthermore, previous studies reported that ROS levels vary significantly depending on the cell type and hormone receptor status of the cells. Lei et al.^34^ showed that low doses of BPF elevates ROS levels, induces cell proliferation, and exerts estrogenic activity by interactions between ERα and GPER1 pathways.

The alteration of homeostasis (oxidant/antioxidant imbalance and cell membrane damage) can lead to potentially severe or permanent effects, especially if exposure occurs during specially sensitive periods of life such as fetal development, infancy and puberty^35^.

Our results in offspring showed a significant reduction of all antioxidant enzymes (CAT, SOD, GPx, GR and GST) in both sexes after LBPF perinatal exposure. Regarding the glutathione system, there was a much more drastic reduction of GSH content in female offspring, leading to a higher GSSG/GSH ratio compared to male offspring. Thus, female offspring showed higher levels of GSSG and thus a greater imbalance in the glutathione system.

Lipid membrane damage occurred in both sexes and levels varied depending on whether measured directly (LPO) or indirectly (MDA, TBARs). HBPF exposure effects on all these parameters were not as noticeable, except for decreased SOD activity in males.

One of the previous studies showed that perinatal exposure to BPF was associated with oxidative damage and metabolic disorders in livers of male mouse offspring. Pregnant mice (F0) were orally gavaged with BPF (100 ng/g b.w./day) from gestational day (GD) 7 to postnatal day (PND) 21. Male offspring in each treatment group were provided a normal diet for 10 weeks after weaning at day 21. Nevertheless, BPF exposure significantly reduced CAT and GSH levels, suggesting disturbances in the antioxidant defense system. Moreover, BPF exposure led to metabolic disorders in the liver due to changes observed in the levels of 8 key metabolites^20^ and lipid accumulation inducing liver damage after perinatal exposure to BPF. Musachio et al.^36^ found sex-specific changes in oxidative stress parameters in *Drosophila melanogaster* after BPF exposure for seven days. Female flies were more susceptible to oxidative cell damage and reduced its longevity. Male flies had higher antioxidant defenses that responded primarily to lower concentrations of BPF, minimizing oxidative cell damage. Perinatal exposure to BPF and analogues affected the body weight of certain organs in male rat offspring and the ovarian function in females. Therefore, BPF can induce reproductive toxicity at low doses^37^. There are few studies in the literature showing BPF effects on offspring, but there is an absolute need to study exposure occurring during early development. The prenatal period is a critical window^38^, where exposure to exogenous compounds such as BPF can affect fetal development. The fetus is extremely vulnerable with limited capacity to metabolize and process such chemicals^39^. In this sense, hepatic UGT2B1 (UDP-glucuronosyltransferase) activity towards BPF in rats is very weak in the fetus and newborn pups^40^. Perinatal exposure and placental transfer (in rats occurs in late gestation)^14^ may also result in developmental tissue changes that contribute to adverse health outcomes in adulthood^41^.

When assessing the adverse effects of bisphenols and analogues, adult organisms and the perinatal effect on offspring have to be taken into account, as they may be affected differently due to different time windows and vulnerability. In turn, when studying the effect on both sexes, even under similar exposure conditions, results in differences due to variability in metabolism, storage and excretion of xenobiotics^42^. It is also important to study different animal models; for example, studies in rodents observed a higher vulnerability to the effects of BPA and other analogues in females than in males^43,44^. Furthermore, the effect at different doses is also important. Thus, according to Vanderberg et al.^45^, more intense low dose effects are common in studies of natural hormones and endocrine disruptors. When non-monotonic dose–response curves are produced, low-dose effects cannot be predicted from the effects observed at high doses. In our results, the maximum effects on antioxidant enzyme involvement, glutathione system and cell membrane damage were observed after exposure to LBPF both in lactating dams and in offspring. This could be explained by the endocrine system responding to very low concentrations of hormones, allowing a maximal biological response without high receptor occupancy of this response or, alternatively, that response mechanisms become saturated before all receptors are occupied. Although the process is very difficult to interpret, the fact is that it has been found by many other authors^46–48^ showing a non-monotonic dose–response manner.

The fact that low doses of BPF produce more noticeable effects than high doses highlights the need for further research to really elucidate the effects of low and environmentally relevant doses both in adulthood and after perinatal exposure to this chemical.

## Conclusions

BPF is one of the most widely used alternatives to BPA in everyday plastic products, resulting that the population is submitted to a constant exposure to low doses of this chemical. In this study, it was observed that exposure to low doses of BPF in female Long Evans rats during pregnancy and lactation increased oxidative stress levels by decreasing the activity of antioxidant enzymes and altering the glutathione system in the liver, the organ responsible for detoxification. This excess of ROS affected cell membranes of hepatocytes, increasing the levels of lipid peroxidation. Similar effects in lactating dams were found in female and male offspring after BPF perinatal exposure. The period of exposure is very important because even if it occurs during the fetal or neonatal period, the effects can influence tissue development and affect adult life. Regardless, further research is needed to elucidate the health risk of BPF exposure in adult life and in offspring.

## Methods

### Chemical and animals

BPF with purity > 99% was purchased from Sigma Aldrich (Switzerland) (CAS Number 620–92-8; article number: 239658). It was dissolved in ethanol and then corn oil at a ratio of 10% ethanol and 90% corn oil. The chosen chow corresponds to a diet with natural ingredients low in phytoestrogens and was purchased from Granovit (Granovit AG, Kaiseraugust, Switzerland).

Thirty-six female (eight weeks of age) and eighteen male (ten weeks of age) Long Evans rats (Janvier Labs, Le Genest-Saint-Isle, France) were housed and maintained in a well-ventilated room at 22 ± 2 °C, with automatic light cycles (12-h light/dark) and all had free access to diet and drinking water ad libitum. Rats were housed in special polypropylene cages (Sodispan Research, Coslada, Madrid) and water bottles were made of glass. A cylindrical environmental enrichment element was included.

The study was approved by the Ethical Committee of Complutense University of Madrid (Madrid, Spain) in accordance with the Guidelines for Ethical Care of Experimental Animals of the European Union (2010/63/UE). This research is within a European project entitled "Novel Testing Strategies for Endocrine Disruptors in the Context of Developmental NeuroToxicity" supported by the European Union's Horizon 2020 Research and Innovation Programme (ENDpoiNTs project; grant number: 825759). All authors complied with the ARRIVE guidelines.

### Experimental design

Animals were randomly divided into three groups: (1) Control (non-treated) group, received chow with a corresponding concentration of corn oil (n = 12 females; n = 6 males) which is the solvent used for the inclusion of the two Bisphenol F dosages in the other groups; (2) Bisphenol F, low dose group (LBPF)—diet intake of 0.0365 mg/kg body weight/day of BPF (n = 12 females; n = 6 males); (3) Bisphenol F, high dose group (HBPF)—diet intake of 3.65 mg/kg body weight/day of BPF (n = 12 females; n = 6 males). The doses of BPF used were chosen according to previous studies on BPA^21,49^ and the large existing literature, where the dose range of BPA (2.5 mg/kg—50 mg/kg) induced impairment learning and memory loss in rodents when BPA was administered in the perinatal period. Thus, the high dose is 3.65 mg/kg higher than 2.5 mg/kg; while the low dose was 100 times lower, to investigate whether, even with such a small dose, any effects were observed.

During premating, female and male rats were treated with the diet with their corresponding dose of BPF for two weeks. Control animals received the control diet for the same time. Mating phase took place between a male and a female from the same group, after checking by vaginal smear that the female was in the estrus phase. The following morning, a check for sperm-positive vaginal smear or sperm-plug was carried out and the process was repeated all mornings for ten days. Dietary treatment was maintained during pregnancy. Six females were pregnant in control and LBPF groups and ten females were pregnant in HBPF group. After birth, the lactating dams were kept in individual cages with their offspring and dietary treatment continued until postnatal day 6 (PND6). PND6 was chosen because the hippocampus is in full development, and they are within the critical period of sexual differentiation of the brain, related to the brain studies on which the ENDpoiNTs project is focused. During the entire experiment (Premating, Mating, Pregnancy, Lactation), the control group cages were kept separately from the BPF-treated groups, to avoid any chance of spreading chow containing BPF that could contaminate it.

**Figure 4:**
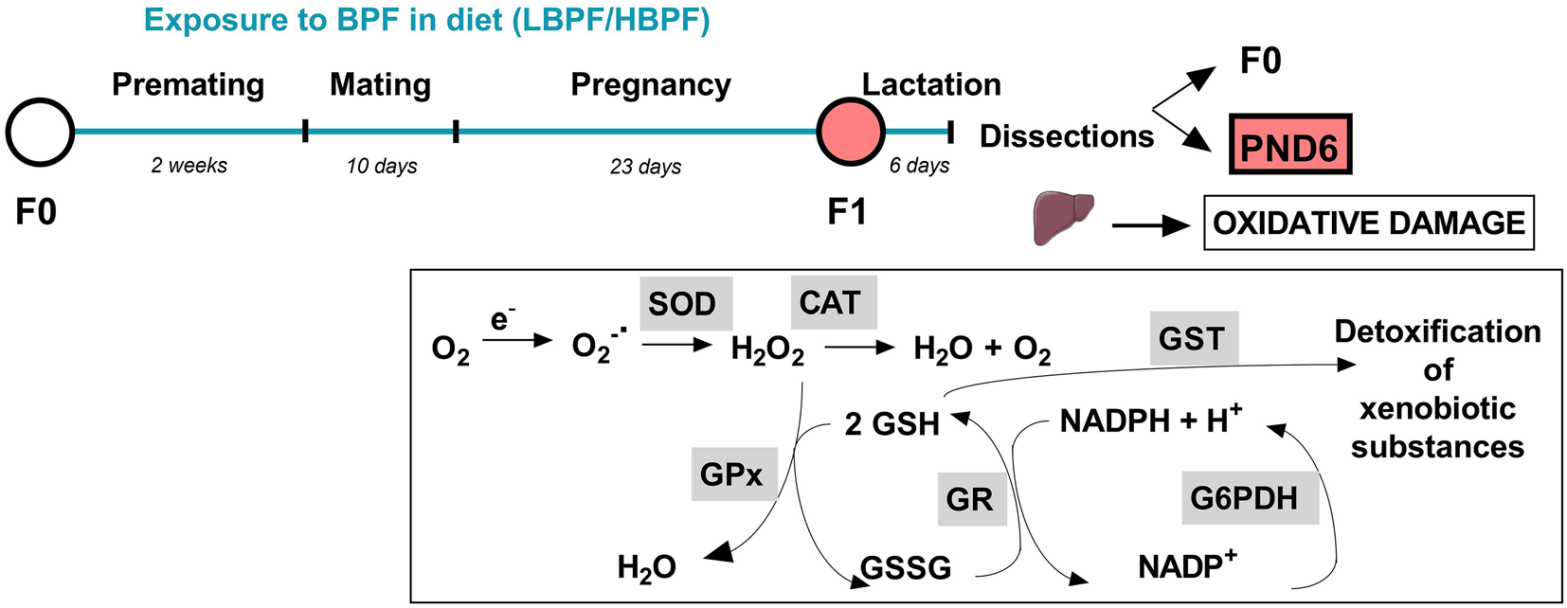
Experimental design. Female and male Long Evans rats (F0) were exposed to a low or high dose of BPF (LBPF or HBPF) or control diet from premating until the end of the experiment. Six days after birth and lactation of the pups, lactating dams (F0) and PND6 pups (females and males) were sacrificed to study the oxidant/antioxidant balance in the liver. Endogenous antioxidant agents include enzymes such as superoxide dismutase (SOD), which eliminates the first oxygen free radical (O_2_) produced during oxygen utilization, the superoxide anion (O_2_^−^.); catalase (CAT), which is responsible for converting hydrogen peroxide (H_2_O_2_) into water (H_2_O) and oxygen (O_2_); and the main enzymes involved in the glutathione system, glutathione peroxidase (GPx), which catalyzes the elimination of peroxides, such as hydrogen peroxide (H_2_O_2_), using reduced glutathione (GSH) and converting it to oxidized glutathione (GSSG), glutathione reductase (GR), which recomposes GSH so that it can be used by GPx, and also glutathione-S-transferase (GST), which catalyzes the conjugation of glutathione with xenobiotics, playing a role in the inactivation of free radicals. Figure created with Prism v7 (GraphPad Software, Inc, CA, USA).

Lactating dams were sacrificed by decapitation using a guillotine. Female and male offspring were sacrificed at PND6 by decapitation using scissors. The livers were collected and immediately frozen in liquid nitrogen and stored at -80 °C until analysis (Fig. 4).

### Activities of antioxidant enzymes

Catalase (CAT), superoxide dismutase (SOD), glutathione peroxidase (GPx), glutathione reductase (GR) and glutathione-S-transferase (GST) activities were measured in the liver homogenate previously lysed with the corresponding buffer and analyzed spectrophotometrically according to the manufacturer's instructions (Cayman Chemical; Ann Arbor, MI, USA).

CAT activity was determined by the reaction with methanol in the presence of an optimal concentration of H2O2. The formaldehyde produced was measured spectrophotometrically with 4-amino-3-hydrazino-5-mercapto-1,2,4-triazole as the chromogen at 540 nm.

SOD activity was assessed by measuring the dismutation of superoxide radicals generated by xanthine oxidase and hypoxanthine. The standard curve generated using this enzyme provides a means to accurately quantify the activity of all three types of SOD (Cu/Zn, Mn, and FeSOD).

GPx activity was measured spectrophotometrically; it was coupled to the oxidation of NADPH by GR. Oxidized glutathione (GSSG), produced upon reduction of an organic hydroperoxide by GPx, is recycled to its reduced state by GR and NADPH. The oxidation of NADPH to NADP + is accompanied by a decrease in absorbance at 340 nm. The rate of decrease in the A340 is directly proportional to the GPx activity in the sample.

GR assay kit was used to measure activity of this enzyme by quantifying the rate of NADPH oxidation. The oxidation of NADPH to NADP + is accompanied by a decrease in absorbance at 340 nm. The rate of decrease in the A340 is directly proportional to the GR activity in the sample.

GST activity was determined spectrophotometrically by measuring formation of the conjugate of reduced glutathione (GSH) and 1-chloro-2,4-dinitrobenzene (CDNB) at 340 nm.

Each sample was tested in duplicate. Enzyme activities were normalized according to liver protein content and were expressed as nmol/min/mg of protein except for SOD which was expressed in U/mg of protein.

### Glutathione concentrations

Liver was homogenized in phosphate buffer 50 mM and EDTA 0.1 M, pH 8. Then, 10 µl of HClO_4_ was added per mL of homogenate and supernatants were used for the quantification of both reduced (GSH) and oxidized (GSSG) glutathione by o-phthalaldehyde (OPT) at pH 12 and pH 8, respectively, resulting in the formation of a fluorescent compound. Fluorescence was measured at 350 nm excitation and 420 nm emission. Results were expressed as nmol of GSSH and GSH per milligram of protein. Moreover, the GSSG/GSH ratio was calculated for each sample.

### Lipid peroxidation determination

Quantification of lipid peroxidation (LPO) was carried out in liver homogenate according to the manufacturer's instructions (Cayman Chemical; Ann Arbor, MI, USA). Lipid Hydroperoxide Assay Kit measures the hydroperoxides directly utilizing the redox reactions with ferrous ions. The amount of lipid hydroperoxide was obtained from the linear regression of the standard curve substituting corrected absorbance values for each sample. LPO content was expressed as nmol/mg of tissue. This procedure eliminates any interference caused by hydrogen peroxide or endogenous ferric ions in the sample and provides a more sensitive and reliable assay for lipid peroxidation.

Lipid peroxidation was also evaluated using the traditional Thiobarbituric acid reactive substances (TBARS) assay. The commercial kit (BioVision, Mountain View, CA, USA) measures the reaction of malondialdehyde (MDA) with thiobarbituric acid (TBA) and the MDA-TBA adduct formation. Samples were resuspended in lysis buffer with the antioxidant butylated hydroxy-toluene (BHT) (0.1 mM) to prevent further formation of MDA during the preparation of the sample or during the heating step. Then, they were centrifuged at 3200 G for 30 min. 200 μL of supernatants from each sample were added to 600 μL TBA, and incubated at 95 °C for 60 min. Samples were cooled in ice for 10 min, and 300 μL of n-butanol were added (Sigma-Aldrich, Madrid, Spain) to create an organic phase in which the MDA molecules were to be placed. Samples were centrifuged and 200 μL of upper organic phase were collected and dispensed into a 96-well microplate for spectrophotometric measurement at 532 nm. Results were expressed as nmol TBARS/mg protein.

### Determination of protein concentration

The protein content of the same samples was evaluated following a bicinchoninic acid protein assay kit protocol (Sigma-Aldrich, Madrid, Spain) using a BSA standard curve.

### Statistical analysis

Results are expressed as the mean ± SD. Mean comparison was done by one-way analysis of variance (ANOVA) followed by the Tukey–Kramer multiple comparison test after testing for normal distribution. A confidence level of 95% (p < 0.05) was considered statistically significant. Statistics were calculated using Prism v7 (GraphPad Software, Inc, CA, USA).

## Data Availability

All data generated or analysed during this study are included in this published article.
